# An automated framework for hypotheses generation using literature

**DOI:** 10.1186/1756-0381-5-13

**Published:** 2012-08-29

**Authors:** Vida Abedi, Ramin Zand, Mohammed Yeasin, Fazle Elahi Faisal

**Affiliations:** 1 Department of Electrical and Computer Engineering, Memphis University, Memphis, TN, 38152, USA; 2 College of Arts and Sciences, Bioinformatics Program, Memphis University, Memphis, TN, 38152, USA; 3 Department of Neurology, University of Tennessee Health Science Center, Memphis, TN, 38163, USA

**Keywords:** Disease network, Disease model, Biological literature-mining, Hypothesis generation, Knowledge discovery, MeSH ontology

## Abstract

**Background:**

In bio-medicine, exploratory studies and hypothesis generation often begin with researching existing literature to identify a set of factors and their association with diseases, phenotypes, or biological processes. Many scientists are overwhelmed by the sheer volume of literature on a disease when they plan to generate a new hypothesis or study a biological phenomenon. The situation is even worse for junior investigators who often find it difficult to formulate new hypotheses or, more importantly, corroborate if their hypothesis is consistent with existing literature. It is a daunting task to be abreast with so much being published and also remember all combinations of direct and indirect associations. Fortunately there is a growing trend of using literature mining and knowledge discovery tools in biomedical research. However, there is still a large gap between the huge amount of effort and resources invested in disease research and the little effort in harvesting the published knowledge. The proposed hypothesis generation framework (HGF) finds “crisp semantic associations” among entities of interest - that is a step towards bridging such gaps.

**Methodology:**

The proposed HGF shares similar end goals like the SWAN but are more holistic in nature and was designed and implemented using scalable and efficient computational models of disease-disease interaction. The integration of mapping ontologies with latent semantic analysis is critical in capturing domain specific direct and indirect “crisp” associations, and making assertions about entities (such as disease X is associated with a set of factors Z).

**Results:**

Pilot studies were performed using two diseases. A comparative analysis of the computed “associations” and “assertions” with curated expert knowledge was performed to validate the results. It was observed that the HGF is able to capture “crisp” direct and indirect associations, and provide knowledge discovery on demand.

**Conclusions:**

The proposed framework is fast, efficient, and robust in generating new hypotheses to identify factors associated with a disease. A full integrated Web service application is being developed for wide dissemination of the HGF. A large-scale study by the domain experts and associated researchers is underway to validate the associations and assertions computed by the HGF.

## Background

The explosion of OMICS*-*based technologies, such as genomics, proteomics, and pharmaco-genomics, has generated a wave of information retrieval tools, such as SWAN [1], to mine the heterogeneous, high dimensional and large databases, as well as complex biological networks. The general characteristics of such complex systems as well as their robustness and dynamical properties were reported by many researchers (i.e., [2,3]). These reports of designing scalable and efficient knowledge discovery tools can further our understanding of complex biological systems. The burgeoning gap between the effort and investment made to acquire the knowledge about complexities of biological systems is disproportionately large compared to the development of knowledge discovery tools that can be used for effectively disseminating the acquired knowledge, generating and validating hypothesis, and understanding the complex causal relationships. Despite a plethora of efforts in reverse-engineering of complex systems to predict response to perturbations, there is a lack of significant effort to create a higher level abstraction of such complex biological systems using sources of information other than genetics data [2,4]. A high level view of complex systems would be very useful in generating new hypotheses and connecting seemingly unrelated entities. Such an abstraction could facilitate translational research and may prove vital in clinical studies by providing a valuable reference to the clinicians, researchers, and other domain experts.

Disease networks can provide a high level view of complex systems; however, the reported networks are mostly based on genetic and proteomic data [2,4]. Such networks could also be constructed based on literature data to incorporate a wider range of factors such as side effects and risk factors. Generating disease-models based on literature data is a very natural and efficient way to better understand and summarize the current knowledge about different high-level systems. A connection between two diseases can be formalized by risk factors, symptoms, treatment options, or any other diseases as compared to only common disease-genes. The relations between diseases can provide a systematic approach to identify missing links and potential associations. It will also create new avenues for collaborations and interdisciplinary research.

To construct a disease network based on literature data, it is imperative to have a scalable and efficient literature-mining tool to explore the huge textual resources. Nevertheless, mining of biological and medical literature is a very challenging task [5-7]. This can further be complicated by challenges with the implementation of relevant information extraction, also known as deep parsing, which is built on formal mathematical models. Deep parsing, also known as formal grammar, attempts to describe how text is generated in the human mind [5]. Deterministic or probabilistic context-free grammars are probably the most popular formal grammars [7]. Grammar-based information extraction techniques are computationally expensive as they require the evaluation of alternative ways to generate the same sentence. Grammar-based information could therefore be more precise but at the cost of reduced processing speed [5].

An alternative to the grammar-based methods are factorization methods such as Latent Semantic Analysis (LSA) [8], and Non-negative Matrix Factorization (NMF) [9,10]. Factorization methods rely on bag-of-word concept, and have therefore reduced computational complexity. LSA is a well known information retrieval technique which has been applied to many areas in bioinformatics. Arguably, LSA captures semantic relations between various concepts based on their distance in the reduced *eigen* space [11]. It has the advantage of extracting direct and indirect associations between entities. A commonly used distance measure in LSA is the cosine value of the angle between the document and query in the reduced *eigen* space.

Over the past two decades, medical text-mining has proved to be valuable in generating new exciting hypotheses. For instance, titles from MEDLINE were used to make connections between disconnected arguments: 1) the connection between migraine and magnesium deficiency [12] which has been verified experimentally; 2) between indomethacin and Alzheimer’s disease [12]; and finally 3) between *Curcuma longa* and retinal diseases [13]. Hypothesis generation in literature-mining relies on the fact that chance connections can emerge to be meaningful [7].

This paper designs and implements an efficient and scalable literature-mining framework to generate and also validate plausible hypotheses about various entities that include (but not limited to): risk factors, environmental factors, lifestyle, diseases, and disease groups. The proposed hypothesis generation framework (HGF) is implemented based on parameter optimized latent semantic analysis (POLSA) [14] and is suitable to capture direct and indirect association among concepts. It is easy to note that the overall performance and quality of results obtained through LSA-based systems is a function of the dictionary used. The concept of mapping ontologies was integrated with the POLSA to overcome such limitations and to provide crisp associations. In particular, the Medical Subject Headings (MESH) is used to construct the dictionary. Such a dictionary allows a more efficient use of the LSA technique in finding semantically related entities in the biological and medical sciences. This framework can be used to generate customized disease-disease interaction networks, to facilitate interdisciplinary collaborations between scientists and organizations, to discover hidden knowledge, and to spawn new research directions. In addition, the concept of statistical disease modeling was introduced to compute the strongly related, related, and not related concepts.

The following section describes the proposed hypothesis generation framework and its evaluation. Two case studies were performed to showcase the potential and utility of the proposed method. Finally, the paper ends with a brief conclusion and discussions about the strengths and weaknesses of the method.

## Results and discussion

### Hypotheses generation framework (HGF)

**Figure 1:**
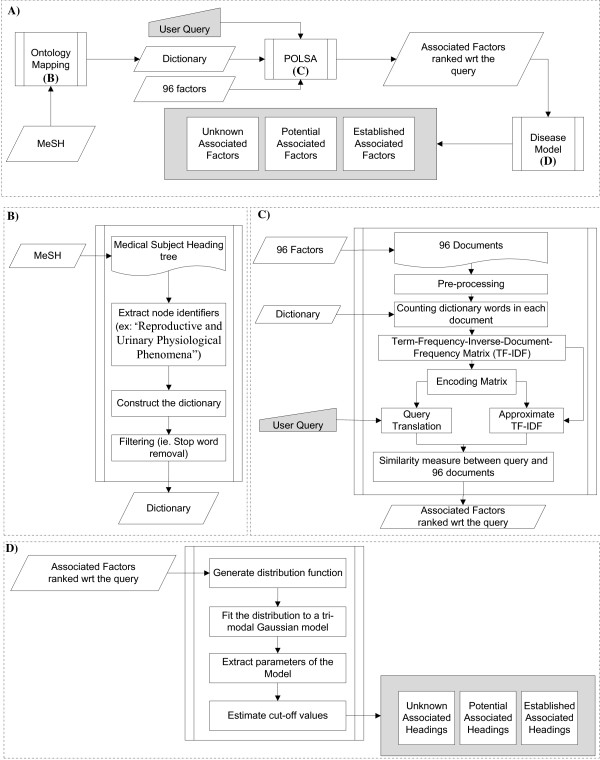
**Flow diagram of the hypothesis generation framework (HGF).A**) In a medical and biological setting, Ontology Mapping could use the Medical Subject Heading (MeSH) and generate a context specific dictionary, which is one of the parameters of the POLSA model. Associated factors are ranked based on a User Query which can be any word(s) in the dictionary. These factors are subsequently grouped into three different bins (unknown factors, potential factors or established factors) based on our Disease Model. **B**) Ontology Mapping to create domain specific dictionary. **C**) Parameter Optimized Latent Semantic Analysis Module. **D**) Disease Model Module.

The HGF has three major modules: Ontology Mapping to generate data-driven domain specific dictionaries, a parameter optimized latent semantic analysis (POLSA), and Disease Model. The schematic diagram of the overall HGF framework is shown in the Figure 1(A). The model is constructed using the POLSA framework, and it is based on the selected documents and the dictionary (Figure 1C). Users can query the model and the output is a ranked list of headings. These ranked headings are grouped into three sets (unknown factors, potential factors, or established factors) using the Disease Model module (Figure 1C and 1D). Analyzing the headings in the three sets can facilitate hypothesis generation and information retrieval based on user query.

#### Ontology mapping

MeSH is used to generate the dictionary in the POLSA model. The mapping of MeSH ontology to create the dictionary for the POLSA significantly enhances the quality of results and provides a crisp association of semantically related entities in biological and medical science. All MeSH headings are reduced to single words to create the context specific and data driven dictionary (see Figure 1B). For instance, “Reproductive and Urinary Physiological Phenomena” is a MeSH term and is reduced to five words in the dictionary (1. Reproductive, 2. and, 3. Urinary, 4. Physiological, and 5. Phenomena). In the filtering step, duplicates as well as stop words such as “and” or words containing fewer than three characters are removed. The final size of this dictionary is 19,165 words. Any dictionary word could be used as a query to the HGF. For instance, the disease “stroke” is a query in this study. The highly ranked factors with respect to a query-disease are considered factors associated with that disease. Cosine similarity measure is used as a metric in the HGF.

#### POLSA module

In order to develop an effective literature-mining framework to model disease-disease interaction networks, generate plausible new hypotheses, and support knowledge-discovery by finding semantically related entities, a Parameter Optimized LSA (POLSA) [14] was re-designed and adopted in the proposed HGF framework.

In addition, a set of associated factors was selected to represent interaction between diseases. Ninety-six common associated factors (see Table 1) were selected through a literature review from numerous medical articles by two domain experts. As the first step, a set of articles was selected by querying the PubMed database using a series of diseases and factors. In the second step, the retrieved articles were manually reviewed by domain experts and entities that were associated with diseases or factors were selected. All articles considered for this analysis were peer reviewed articles. In addition, some common diseases such as diabetes and depression were also included in the set of 96 factors, as these are believed to be, in many instances, risk factors to other diseases. Therefore, the set of 96 associated factors represents a wide range of factors including generic factors such as depression and infection as well as specific factors such as vitamin E. As the final step, the set was further revised by an expert in the medical field. Using the improved POLSA technique [14], meaningful associations from the textual data in the PubMed database are extracted and mined. Furthermore, the factors are ranked based on their level of association to a given query.

**Table 1:** Potential risk factors and/or contributing factors selected by medical expert

***Potential contributing factors***	***Categorys***
Asthma, autism, schizophrenia, HIV, immunological disorder, bipolar, hypertension, osteoporosis, coronary heart disease (CHD), diabetes, allergy, herpes, leukemia, breast cancer, lymphoma, hypothyroidism, hyperthyroidism, insomnia, depression, viral infection, bacterial infection, hepatitis B virus, retrovirus, enterovirus	Disease / medical condition
morning cortisol level, cholesterol level, head trauma, abdominal adiposity, fracture, bone mineral density (BMD), body mass index (BMI), pregnancy outcome, maternal influenza, postmenopause, mood, volume of cerebrum, volume of hippocampus, volume of lateral ventricle, family history, motor activity assessment	Sign / symptom
caffeine, hormone, aflatoxin, calcium deficiency or calcium overdose, phosphorus deficiency or phosphorus overdose, magnesium deficiency or magnesium overdose, sodium deficiency or sodium overdose, potassium deficiency or potassium overdose, sulphur deficiency or sulphur overdose, chloride deficiency or chloride overdose, chromium deficiency or chromium overdose, copper deficiency or copper overdose, fluoride deficiency or fluoride overdose, iodine deficiency or iodine overdose, iron deficiency or iron overdose, manganese deficiency or manganese overdose, molybdenum deficiency or molybdenum overdose, selenium deficiency or selenium overdose, zinc deficiency or zinc overdose, vitamin A or Retinol, vitamin B1 or Thiamine, vitamin B2 or Riboflavin, vitamin B3 or Niacin, vitamin B5 or Pantothenic acid, vitamin B6 or Pyridoxine, vitamin B7 or Biotin, vitamin, B9 or Folic acid, vitamin B12 or Cyanocobalamin, vitamin C or Ascorbic acid, vitamin D or Calciferol, vitamin E or Tocopherol, vitamin K or Phylloquinone, Cannabis, cocaine, bisphenol-A (PBA), diethylstilbestrol (DES), estradiol (E2), oral contraceptive (OC)	Chemical compound
air pollutants, volatile organic compounds, Pesticide, chemical agents, wood dust (exposure), silica dust (exposure), night shift work, outdoor workers, indoor workers, exposure polycyclic aromatic hydrocarbons, heterosexual, homosexual, Tobacco smoking, alcohol consumption, health education and health promotion, addiction, lifestyle intervention, diet nutrition, stress, age gender, breast-feeding	Environmental / life style and behavioral factors

Titles and abstracts from PubMed (for the past twenty years) for each of the 96 factors were downloaded in a local machine. On average there were 47,570 abstracts per factor; the specific factors such as “maternal influenza” had fewer abstracts associated with them (minimum of 160 abstracts/factor) and the more generic factors such as “hormone” were associated with a greater number of abstracts (a maximum of 557,554 abstracts/factor). The complete collection was then used to construct the knowledge space for the POLSA model. Using a query such as “Parkinson” or “stroke” the 96 factors were then ranked based on their relative level of associations to the query. The distribution of a set of associated factors with respect to a disease was modeled as a tri-modal distribution: a distribution which has three modes. This is due to the fact that some factors are known to be associated with the disease and have high scores. Similarly, some factors are known to be unassociated to the disease and these have negative scores; in addition, some factors may or may not be associated to the disease and these have low similarity scores. Matlab was used to generate two tri-modal distributions based on general Gaussian models for the two distributions obtained from queries “stroke” and “Parkinson”. The model uses the following formulation to describe the tri-modal Gaussian distribution:

(1) $$f=\alpha_{1}*\exp\left\{-{\left(\frac{\left(x-\mu_{1}\right)}{\sigma_{1}}\right)}^{2}\right\}+\alpha_{2}*\exp\left\{-{\left(\frac{\left(x-\mu_{2}\right)}{\sigma_{2}}\right)}^{2}\right\}+\alpha_{3}*\exp\left\{-{\left(\frac{\left(x-\mu_{3}\right)}{\sigma_{3}}\right)}^{2}\right\}\text{,}$$

Where α_1_, α_2_ and α_3_ are the scaling factors; μ_1_, μ_2_ and μ_3_ are the position of the center of the peaks, and σ_1_, σ_2_, σ_3_ control the width of the distributions. The goodness of fit was measured using an R-square score.

#### Disease model

**Figure 2:**
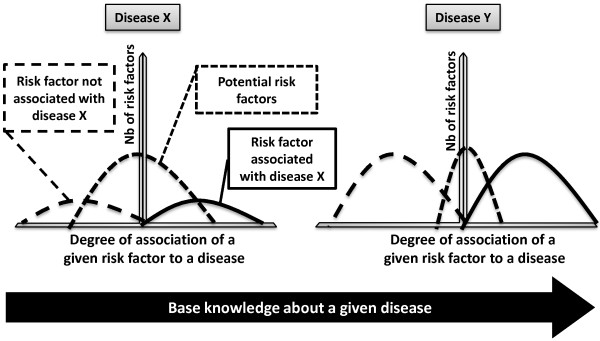
**Model for the distribution of associated factors of a given disease.** If associated factors – such as risk factors – of a disease are well known as in the case for Disease Y, then the two dominating distributions are the factors that are associated and those that are not associated with the disease; if on other hand the associated factors of a disease are not well documented (Disease X) then the dominating distribution is that of the potential factors.

Using a disease model (see Figure 2), it was possible to map the mixture of three Gaussian distributions into easy to understandable categories. The implicit assumption is that if associated factors of a disease are well known, a large body of literature will be available to corroborate the existence of such associations. On the other hand, if associated factors of a disease are not well documented, the factors are weakly associated to the disease with few factors displaying a high level of association (Disease X versus Disease Y as shown in the Figure 2). Since the distribution of association level of factors (including risk factors) will be different in the two scenarios. In the first case (Disease Y) the two dominating distributions are the factors that are associated and those that are not associated with the disease; in the second case (Disease X) the dominating distribution is that of the potential factors. In essence, if one accepts this assumption then the distribution of associated factors follows a tri-modal distribution and it will be intuitive to measure the level of association for different factors with respect to a given disease. Utilization of a disease model (by a tri-modal distribution) allows better identification of the three sets of factors: unknown associations, potential associations and established associations.

Separating the three distributions allows implementation of a dynamic and data-driven threshold calculation. Hence, the parameters of the distributions can be used to model a cut-off threshold for the factors that are established, potential, or unknown. This method is empirical and provides an intuitive approach to evaluate the results. The score can be further optimized in a heuristic manner with utilization of a large-scale and comprehensive ground truth set. Furthermore, the highly associated factors to the disease are the well known factors; the hidden knowledge on the other hand resides in the region where the associations are positive yet weak.

### Model evaluation

**Figure 3:**
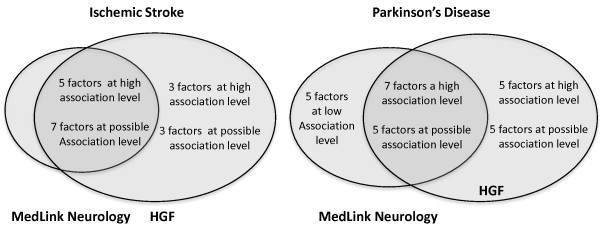
**Number of factors identified by MedLink Neurology and by HGF for IS and PD.** Association levels for IS measured by HGF are high (0.3 < cosine score) and possible (0.1 < cosine score < 0.3); association levels for PD measured by HGF are high (0.2 < cosine score), possible (0.1 < cosine score < 0.2) or low (0.05 < cosine score < 0.1).

**Figure 4:**
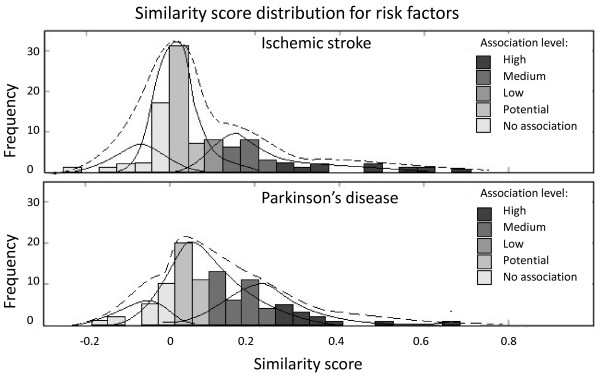
**Distribution of similarity score (dashed line) for risk factors associated with IS and PD.** The frequency represents the number of factors at each cosine similarity level (−1 to +1). Tri-modal distribution models are represented by solid lines.

Two diseases, namely, Ischemic Stroke (IS) and Parkinson’s Disease (PD), were used as queries to the hypothesis generation system. The distribution of associated factors is presented in the Figure 3. The results were compared with MedLink neurology [15], a web resource used by clinicians. Comparative results were summarized in the Figure 4. In the case of IS, most of the associated factors are identified by both systems; however there is a set of factors that have only been identified by the proposed approach. In the case of the PD, a large number of factors have been identified by both systems. However, there are a number of factors that have only been identified by the proposed HGF and only a handful that are mentioned in the MedLink neurology which have positive but low similarity score in the hypothesis generation framework.

The tri-modal distribution model is used to group the associated factors into different levels. The cut-off values to differentiate between different association levels vary slightly depending on the distribution of the similarity scores. The ideal decision boundary can be found if a large number of ground truth cases are available; in this situation the decision boundary is selected intuitively based on the shape of the distributions. For example, in the case of IS, factors are considered highly associated if their cosine score is greater than 0.3, factors are possible associated if their score is between 0.1 and 0.3 and are possibly not associated if their score is lower than 0.1. In the case of PD, factors are considered highly associated if their cosine score is greater than 0.2, factors are possibly associated if their cosine score is between 0.1 and 0.2 and finally the factors with scores between 0.05 and 0.1 are considered associated at low level, factors with scores lower than 0.05 are considered possibly not associated with the Parkinson’s Disease.

In the case of IS, the distribution of known associated factors are more shifted to the right as compared to the factors in PD, hence the separation between the known and unknown factors is more pronounced. In addition to that, associations at both extreme levels (close to +1.0 and −1.0) are likely to be common knowledge; however, the hidden knowledge tends to be captured at similarity scores that are low yet positive. Nonetheless, it is not realistic to compare the precise similarity score values in order to give more importance to one factor versus another factor mainly because there is a systemic bias that is inherent to the biological text data and causes the generic factors to be an underestimate of the true value (data not shown); hence a direct comparison would fail in this case if no additional normalization steps are taken.

Figure 3 summarizes a comparative analysis of MedLink Neurology and HGF for IS and PD. Overall in the case of IS, twelve factors were identified by both systems and six factors were identified by the HGF. In the case of PD, twelve factors were identified by both systems, ten factors were identified by the HGF and five factors were identified by MedLink Neurology. But, these factors had a low association level in HGF. The five factors were either very generic or were not exactly mapped in the set of the 96 factors, hence a direct comparison could not be made. Finally, this small scale comparative analysis corroborates the hypothesis that HGF based on literature can better predict the associated factors for diseases such as IS when the risk and associated factors are well studied and documented. In both cases, MedLink, Neurology, and HGF predicted twelve common associated factors; however, in the case of PD ten new factors were predicted in comparison to six in the case of IS.

## Discussion

*De novo* hypothesis generation can provide an approach on how we design experiments and select the parameters for the study. Interestingly, associations detected by the proposed framework can facilitate extraction of interesting observations and new trends in the field. For instance, it was found that PD could possibly be associated with immunological disorders; this is an intriguing observation. This analysis also facilitates interdisciplinary research and enhances interaction among scientists from sub-specialized fields. A manual review of the literature is performed to find evidences for some of the associations found only by the HGF; Table 2 summarizes these results.

**Table 2:** A subset of factors identified only by the hypothesis generation framework

***Query***	***Factors***	***Level of association (cosine score)***	***References***
***Ischemic stroke***	Calcium/Minerals	0.13	[16,17]
	Depression (morning cortisol level, mood, stress)	0.48, 0.18, and 0.12	[18,19]
	Vitamin E	0.12	[20]
***Parkinson’s disease***	Immunological disorders	0.29	[21-27]
	Hyperthyroidism	0.1	[28-32]

There are three main limitations in the presented framework. We are currently in the process of finding solutions for these limitations. 1) Manual selection of the factors creates bias in the dataset and also limits its scalability property. To alleviate this problem, MeSH hierarchy will be used to generate the set of factors. MeSH comprises more than 25,000 subjects headings organized in an eleven-level hierarchy. 2) In the set of 96 factors, some factors were very generic and some very specific, therefore, there was a systemic bias in the dataset which caused the score for generic factors to be an underestimate of the true values and factors with limited information to be overestimated (data not shown). To partially solve this technical difficulty, an improved method based on local LSA is being developed in our lab. And finally, 3) looking only at literature from the past twenty years was not sufficient for the HGF. The expansion of the literature is necessary based on the observation that the association between head trauma and PD was significantly lower than expected.

## Conclusion

Generating new hypotheses by mining a vast amount of raw unstructured knowledge from the archived reported literature may help in identifying new research trends as well as promoting interdisciplinary studies. In addition, the presented framework is not limited to uncovering disease-disease interactions; any word from MeSH can be used to query the system, and its associated factors can be identified accordingly. Disease-disease interaction networks, interaction networks among chemical compounds, drug-drug interaction networks, or any specific type of interaction network can be constructed using the HGF. The common basis for all these networks is the knowledge embedded in the literature. Application of this framework is broad as its usage is not limited to any specific domain. For instance, uncovering drug-drug interactions is valuable in drug development and drug administration, uncovering disease-disease interaction is important in understanding disease mechanism’s and advancing biology through integrated interdisciplinary research. Even though the framework is not limited to diseases, in this study two neurological diseases were used to test the system and demonstrate the power and applicability of the framework.

In addition to addressing the limitations of the framework, work is in progress to expand the HGF framework to allow the user to generate disease networks based on a number of user-defined queries. Such customized networks can be valuable to a wide range of scientists by promoting a faster identification of associated factors and detection of disease-disease interactions. Disease networks based on genetics and proteomics data display many connections between individual disorders and disease categories [2,4]. Therefore, as expected each human disorder does not seem to have unique origins or be independent of other disorders. To uncover potential links between two disorders knowledge extraction from medical literature could be greatly beneficial and reliable.

## Abbreviations

HGF: Hypothesis generation framework; IS: Ischemic stroke; LSA: Latent semantic analysis; MeSH: Medical subject heading; NMF: Non-negative matrix factorization; PD: Parkinson’s disease; POLSA: Parameter optimized latent semantic analysis.

## Competing interests

The authors declare that they have no competing interests.

## Authors’ contributions

VA designed and carried out the experiments, participated in the development of the methods, analyzed the results and drafted the manuscript. RZ participated in the development of the methods, designed the validation experiments for the two test cases and reviewed the manuscript. FEF participated in the implementation of the algorithms. MY participated in the development of the methods, supervised the experiments and edited the manuscript. All authors have read, and approved the final version of the manuscript.

## Authors’ information

VA is a Ph.D. candidate in Electrical and Computer Engineering at the University of Memphis; she has a B.A.Sc. in Computer Engineering and B.Sc. in Biochemistry in addition to a M.Sc. in Cellular Molecular Medicine and a second M.Sc. in Bioinformatics. Her research interests are interdisciplinary research in Medical Informatics and Systems Biology. VA’s research incorporates a systems approach to understanding gene regulatory networks, which combines mathematical modeling and molecular biology wet lab techniques. Her recent contributions are in medical informatics where her board understanding of interdisciplinary issues as well as deep knowledge in mathematics and experimental biology are fundamental in designing and performing experiments in translational research.

RZ is a M.D. in the department of Neurology at the University of Tennessee. He also holds a Masters of Public Health. His research interests include Vascular Neurology and Bioinformatics. Over the past few years, RZ has contributed to bridge the gap between clinical findings and application of bioinformatics tools.

FEF is a PhD candidate in Electrical and Computer Engineering at The University of Memphis; he has a B.Sc. in Computer Science and Engineering, M.Sc. degree in Computer Science and Engineering and a second M.Sc. degree in Bioinformatics. His research interests are biological information retrieval and data mining. FEF possesses good knowledge in software design and development. He participated in software development of some national and international research projects, such as Codewitz Asia-Link Project of European Union.

MY is an Associate Professor in the department of Electrical and Computer Engineering, adjunct faculty member of Biomedical Engineering and Bioinformatics Program, and an affiliated member of the Institute for Intelligent Systems (IIS) at The University of Memphis (U of M). He is a senior member of the IEEE. He made significant contributions in the research and development of real-time computer vision solutions for academic research and commercial applications. He has been involved with several technological innovations, including classifying gender, age group, ethnicity and emotion, face detection, recognition of human activities in video, and speech-gesture enabled sophisticated natural human-computer interfaces. Some of his research on facial image analysis and hand gesture recognition is used in developing several commercial products by the Videomining Inc.
